# 2-Bromo-1,3-bis­(4-chloro­phen­yl)prop-2-en-1-one

**DOI:** 10.1107/S160053680903815X

**Published:** 2009-10-03

**Authors:** William T. A. Harrison, Q. N. M. Hakim Al-Arique, B. Narayana, H. S. Yathirajan, B. K. Sarojini

**Affiliations:** a Department of Chemistry, University of Aberdeen, Meston Walk, Aberdeen AB24 3UE, Scotland; b Department of Studies in Chemistry, University of Mysore, Manasagangotri, Mysore 570 006, India; c Department of Chemistry, Mangalore University, Mangalagangotri 574 199, India; d Department of Chemistry, P. A. College of Engineering, Nadupadavu, Mangalore 574 153, India

## Abstract

In the title compound, C_15_H_9_BrCl_2_O, the two benzene rings are twisted from each other with a dihedral angle of 47.33 (8)°. The crystal structure is stabilized by aromatic π–π inter­actions between the benzene rings of neighbouring mol­ecules [centroid–centroid distance = 3.680 (2) Å], and by weak inter­molecular C—H⋯O and C—H⋯Cl inter­actions. Additionally, the crystal structure exhibits a short intra­molecular C—H⋯Br contact (H⋯Br = 2.69 Å).

## Related literature

For background on chalcones as possible nonlinear optical materials, see: Harrison *et al.* (2006 ▶). For related structures with the same backbone and different substituents on the aromatic rings, see: Butcher *et al.* (2006 ▶, 2007 ▶); Dhanasekaran *et al.* (2007*a* ▶,*b* ▶); Fun *et al.* (2008 ▶).
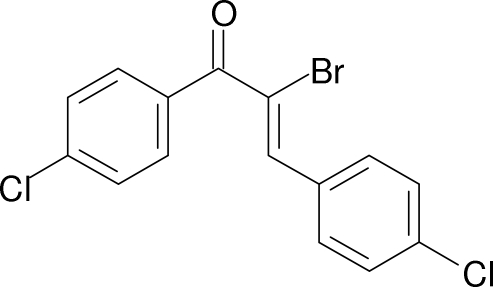


## Experimental

### 

#### Crystal data

C_15_H_9_BrCl_2_O
*M* *_r_* = 356.03
Monoclinic, 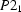

*a* = 7.7416 (3) Å
*b* = 9.7981 (4) Å
*c* = 9.6717 (3) Å
β = 109.075 (2)°
*V* = 693.34 (5) Å^3^
*Z* = 2
Mo *K*α radiation
μ = 3.34 mm^−1^
*T* = 120 K
0.18 × 0.16 × 0.06 mm

#### Data collection

Nonius KappaCCD diffractometer
Absorption correction: multi-scan (*SADABS*; Bruker, 2003 ▶) *T*_min_ = 0.585, *T*_max_ = 0.824
12526 measured reflections
3129 independent reflections
2873 reflections with *I* > 2σ(*I*)
*R*_int_ = 0.040

#### Refinement

*R*[*F* ^2^ > 2σ(*F* ^2^)] = 0.036
*wR*(*F* ^2^) = 0.079
*S* = 1.04
3129 reflections
172 parameters
1 restraint
H-atom parameters constrained
Δρ_max_ = 1.20 e Å^−3^
Δρ_min_ = −0.46 e Å^−3^
Absolute structure: Flack (1983 ▶), 1434 Friedel pairs
Flack parameter: 0.044 (9)

### 

Data collection: *COLLECT* (Nonius, 1998 ▶); cell refinement: *SCALEPACK* (Otwinowski & Minor, 1997 ▶); data reduction: *DENZO* (Otwinowski & Minor, 1997 ▶), *SCALEPACK* and *SORTAV* (Blessing, 1995 ▶); program(s) used to solve structure: *SHELXS97* (Sheldrick, 2008 ▶); program(s) used to refine structure: *SHELXL97* (Sheldrick, 2008 ▶); molecular graphics: *ORTEP-3* (Farrugia, 1997 ▶); software used to prepare material for publication: *SHELXL97*.

## Supplementary Material

Crystal structure: contains datablocks I, global. DOI: 10.1107/S160053680903815X/lx2111sup1.cif

Structure factors: contains datablocks I. DOI: 10.1107/S160053680903815X/lx2111Isup2.hkl

Additional supplementary materials:  crystallographic information; 3D view; checkCIF report

## Figures and Tables

**Table 1:** Selected torsion angles (°)

C6—C7—C8—C9	32.6 (5)
O1—C7—C8—Br1	31.2 (5)
C7—C8—C9—C10	174.4 (4)

**Table 2:** Hydrogen-bond geometry (Å, °)

*D*—H⋯*A*	*D*—H	H⋯*A*	*D*⋯*A*	*D*—H⋯*A*
C1—H1⋯O1^i^	0.95	2.47	3.411 (5)	171
C11—H11⋯Cl1^ii^	0.95	2.81	3.619 (4)	143
C15—H15⋯Br1	0.95	2.69	3.377 (4)	129

Symmetry codes: (i) 
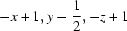
; (ii) 
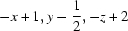
.

**Fig. 1.**
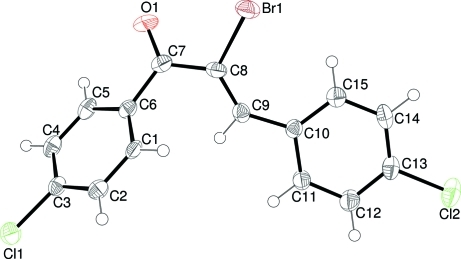
View of the molecular structure of (I) showing 50% displacement ellipsoids. The H atoms are drawn as spheres of arbitrary radius.

## supplementary crystallographic information

### Comment

As part of our ongoing investigations of chalcone derivatives as possible non-linear optical materials (Harrison *et al.*, 2006), we now report the synthesis and structure of the noncentrosymmetric title compound, (I), (Fig 1.).

The molecule adopts a twisted conformation with the dihedral angle between ring A (C1-C6) and ring B (C10-C15) being 47.33 (8)°. Some of the atoms bonded to the benzene rings deviate significantly from their attached ring planes: Cl1 and C7 deviate by 0.106 (5) and 0.140 (6)Å respectively from the mean plane of C1-C6 and Cl2 and C9 deviate by 0.028 (5) and 0.063 (6)Å from the mean plane of C10-C15. The dihedral angles between atoms C7/C8/C9 and ring planes A and B are 55.9 (2) and 20.1 (3)°, respectively. The strongly twisted conformation (Table 1) may arise, in part, to relieve the short intramolecular H1···H9 contact of 2.35 Å. A short intramolecular C–H···Br contact occurs (Table 1).

The crystal packing for (I) is influenced by weak intermolecular C–H···O and C–H···Cl interactions (Table 2), resulting in a noncentrosymmetric structure. The C–H···O links lead to chains propagating in [010], which appear to be reinforced by aromatic π–π stacking between the A and B rings [centroid-centroid separation = 3.680 (2) Å; inter-plane angle = 10.82 (19)°]. The weaker C–H···Cl interaction also generates [010] chains and together, the non-classical bonds lead to (100) sheets.

### Experimental

2,3-Dibromo-1,3-[bis(4-chlorophenyl)]-2-propan-1-one (4.32 g, 0.01 mol) was mixed with triethylamine (5 ml, 0.05 mol) in toluene (100 ml). The mixture was stirred well for 24 hrs and the precipitated ethylenehydrobromide was filtered off and the solvent was removed under reduced pressure. The resulting solid mass obtained on cooling was collected by filtration. The compound was dried and recrystallized four times with ethanol to yield colourless blocks of (I). Yield: 60%; m. p.: 325-328 K; analysis for C_15_H_9_BrCl_2_O: found (calculated): C: 18.01 (18.02); H: 9.15 (9.07).

### Refinement

The H atoms were placed in calculated positions (C–H = 0.95 Å) and refined as riding with *U*_iso_(H) = 1.2*U*_eq_(C). The highest difference peak is 0.96Å from O1.

### Crystal data

**Table:**

C_15_H_9_BrCl_2_O	*F*(000) = 352
*M**_r_* = 356.03	*D*_x_ = 1.705 Mg m^−^^3^
Monoclinic, *P*2_1_	Mo *K*α radiation, λ = 0.71073 Å
Hall symbol: P 2yb	Cell parameters from 13953 reflections
*a* = 7.7416 (3) Å	θ = 2.9–27.5°
*b* = 9.7981 (4) Å	µ = 3.34 mm^−^^1^
*c* = 9.6717 (3) Å	*T* = 120 K
β = 109.075 (2)°	Block, colourless
*V* = 693.34 (5) Å^3^	0.18 × 0.16 × 0.06 mm
*Z* = 2	

### Data collection

**Table:**

Nonius KappaCCD diffractometer	3129 independent reflections
Radiation source: fine-focus sealed tube	2873 reflections with *I* > 2σ(*I*)
graphite	*R*_int_ = 0.040
Detector resolution: 10.0 pixels mm^-1^	θ_max_ = 27.5°, θ_min_ = 3.1°
ω and φ scans	*h* = −10→10
Absorption correction: multi-scan (*SADABS*; Bruker, 2003)	*k* = −12→12
*T*_min_ = 0.585, *T*_max_ = 0.824	*l* = −12→12
12526 measured reflections	

### Refinement

**Table:**

Refinement on *F*^2^	Secondary atom site location: difference Fourier map
Least-squares matrix: full	Hydrogen site location: difference Fourier map
*R*[*F*^2^ > 2σ(*F*^2^)] = 0.036	H-atom parameters constrained
*wR*(*F*^2^) = 0.079	*w* = 1/[σ^2^(*F*_o_^2^) + (0.0258*P*)^2^ + 0.5496*P*] where *P* = (*F*_o_^2^ + 2*F*_c_^2^)/3
*S* = 1.04	(Δ/σ)_max_ < 0.001
3129 reflections	Δρ_max_ = 1.20 e Å^−^^3^
172 parameters	Δρ_min_ = −0.46 e Å^−^^3^
1 restraint	Absolute structure: Flack (1983), 1434 Friedel pairs
Primary atom site location: structure-invariant direct methods	Flack parameter: 0.044 (9)

### Special details

**Table:**

Geometry. All e.s.d.'s (except the e.s.d. in the dihedral angle between two l.s. planes) are estimated using the full covariance matrix. The cell e.s.d.'s are taken into account individually in the estimation of e.s.d.'s in distances, angles and torsion angles; correlations between e.s.d.'s in cell parameters are only used when they are defined by crystal symmetry. An approximate (isotropic) treatment of cell e.s.d.'s is used for estimating e.s.d.'s involving l.s. planes.
Refinement. Refinement of *F*^2^ against ALL reflections. The weighted *R*-factor *wR* and goodness of fit *S* are based on *F*^2^, conventional *R*-factors *R* are based on *F*, with *F* set to zero for negative *F*^2^. The threshold expression of *F*^2^ > σ(*F*^2^) is used only for calculating *R*-factors(gt) *etc*. and is not relevant to the choice of reflections for refinement. *R*-factors based on *F*^2^ are statistically about twice as large as those based on *F*, and *R*- factors based on ALL data will be even larger.

### Fractional atomic coordinates and isotropic or equivalent isotropic displacement parameters (Å^2^)

**Table:**

	*x*	*y*	*z*	*U*_iso_*/*U*_eq_	
C1	0.5669 (5)	0.6271 (3)	0.7407 (4)	0.0255 (8)	
H1	0.5761	0.5621	0.6706	0.031*	
C2	0.6586 (5)	0.6046 (4)	0.8887 (4)	0.0266 (8)	
H2	0.7296	0.5245	0.9206	0.032*	
C3	0.6438 (4)	0.7025 (4)	0.9891 (3)	0.0241 (6)	
C4	0.5385 (5)	0.8181 (4)	0.9458 (4)	0.0288 (8)	
H4	0.5283	0.8825	1.0160	0.035*	
C5	0.4479 (5)	0.8387 (4)	0.7982 (4)	0.0292 (8)	
H5	0.3753	0.9182	0.7670	0.035*	
C6	0.4622 (5)	0.7436 (3)	0.6945 (4)	0.0250 (8)	
C7	0.3742 (5)	0.7800 (4)	0.5351 (4)	0.0277 (8)	
C8	0.3109 (5)	0.6665 (4)	0.4271 (4)	0.0271 (8)	
C9	0.2468 (5)	0.5475 (4)	0.4610 (4)	0.0252 (8)	
H9	0.2575	0.5416	0.5615	0.030*	
C10	0.1645 (5)	0.4256 (4)	0.3785 (4)	0.0237 (7)	
C11	0.1567 (5)	0.3124 (4)	0.4629 (4)	0.0304 (8)	
H11	0.2057	0.3188	0.5665	0.037*	
C12	0.0786 (5)	0.1897 (4)	0.3994 (4)	0.0341 (9)	
H12	0.0774	0.1119	0.4578	0.041*	
C13	0.0028 (5)	0.1849 (4)	0.2478 (4)	0.0303 (8)	
C14	0.0093 (5)	0.2951 (4)	0.1604 (4)	0.0316 (9)	
H14	−0.0427	0.2890	0.0570	0.038*	
C15	0.0928 (5)	0.4146 (4)	0.2258 (4)	0.0276 (8)	
H15	0.1016	0.4899	0.1665	0.033*	
O1	0.3501 (5)	0.8986 (3)	0.4961 (3)	0.0440 (8)	
Cl1	0.76953 (13)	0.67976 (9)	1.17307 (9)	0.0352 (2)	
Cl2	−0.10057 (15)	0.03386 (11)	0.16691 (12)	0.0460 (3)	
Br1	0.31668 (5)	0.71253 (4)	0.23765 (4)	0.03793 (12)	

### Atomic displacement parameters (Å^2^)

**Table:**

	*U*^11^	*U*^22^	*U*^33^	*U*^12^	*U*^13^	*U*^23^
C1	0.0331 (19)	0.0179 (17)	0.0270 (17)	−0.0089 (15)	0.0120 (15)	−0.0038 (14)
C2	0.0268 (18)	0.0234 (18)	0.0321 (19)	0.0006 (15)	0.0130 (15)	−0.0016 (15)
C3	0.0265 (15)	0.0222 (16)	0.0272 (14)	−0.0041 (17)	0.0138 (12)	−0.0040 (17)
C4	0.0303 (19)	0.0246 (18)	0.037 (2)	−0.0052 (16)	0.0185 (16)	−0.0088 (16)
C5	0.0298 (19)	0.0202 (18)	0.040 (2)	−0.0016 (15)	0.0142 (17)	−0.0070 (16)
C6	0.0219 (16)	0.023 (2)	0.0308 (17)	−0.0049 (13)	0.0095 (13)	0.0000 (14)
C7	0.0249 (18)	0.0258 (19)	0.0325 (19)	−0.0014 (15)	0.0094 (15)	0.0036 (16)
C8	0.0251 (17)	0.031 (2)	0.0248 (17)	−0.0008 (14)	0.0068 (14)	0.0095 (14)
C9	0.0223 (17)	0.0268 (19)	0.0232 (16)	0.0026 (15)	0.0028 (14)	−0.0031 (15)
C10	0.0208 (16)	0.0231 (18)	0.0252 (17)	−0.0012 (14)	0.0049 (14)	−0.0020 (14)
C11	0.038 (2)	0.031 (2)	0.0240 (17)	−0.0122 (17)	0.0122 (16)	−0.0030 (16)
C12	0.041 (2)	0.033 (2)	0.0329 (17)	−0.0120 (19)	0.0179 (16)	−0.0028 (18)
C13	0.0268 (17)	0.034 (2)	0.0318 (16)	−0.0077 (16)	0.0124 (14)	−0.0120 (17)
C14	0.0270 (19)	0.038 (2)	0.0254 (18)	0.0029 (16)	0.0022 (15)	−0.0114 (17)
C15	0.0248 (18)	0.0290 (19)	0.0282 (18)	0.0011 (15)	0.0077 (15)	−0.0005 (16)
O1	0.075 (2)	0.0152 (13)	0.0357 (15)	−0.0042 (14)	0.0097 (15)	0.0106 (12)
Cl1	0.0448 (5)	0.0336 (6)	0.0279 (4)	−0.0016 (4)	0.0131 (4)	−0.0049 (4)
Cl2	0.0468 (6)	0.0417 (6)	0.0522 (6)	−0.0183 (5)	0.0199 (5)	−0.0197 (5)
Br1	0.0425 (2)	0.0432 (2)	0.02831 (17)	−0.0063 (2)	0.01189 (14)	0.01032 (19)

### Geometric parameters (Å, °)

**Table:**

C1—C6	1.387 (5)	C8—Br1	1.902 (3)
C1—C2	1.392 (5)	C9—C10	1.462 (5)
C1—H1	0.9500	C9—H9	0.9500
C2—C3	1.397 (5)	C10—C11	1.391 (5)
C2—H2	0.9500	C10—C15	1.402 (5)
C3—C4	1.378 (5)	C11—C12	1.395 (5)
C3—Cl1	1.741 (3)	C11—H11	0.9500
C4—C5	1.384 (5)	C12—C13	1.391 (5)
C4—H4	0.9500	C12—H12	0.9500
C5—C6	1.399 (5)	C13—C14	1.382 (6)
C5—H5	0.9500	C13—Cl2	1.742 (4)
C6—C7	1.510 (5)	C14—C15	1.387 (6)
C7—O1	1.217 (5)	C14—H14	0.9500
C7—C8	1.495 (5)	C15—H15	0.9500
C8—C9	1.349 (5)		
			
C6—C1—C2	120.6 (3)	C7—C8—Br1	113.0 (2)
C6—C1—H1	119.7	C8—C9—C10	134.7 (3)
C2—C1—H1	119.7	C8—C9—H9	112.7
C1—C2—C3	118.4 (3)	C10—C9—H9	112.7
C1—C2—H2	120.8	C11—C10—C15	118.7 (3)
C3—C2—H2	120.8	C11—C10—C9	115.2 (3)
C4—C3—C2	121.8 (3)	C15—C10—C9	126.1 (3)
C4—C3—Cl1	119.6 (3)	C10—C11—C12	121.6 (3)
C2—C3—Cl1	118.5 (3)	C10—C11—H11	119.2
C3—C4—C5	119.0 (3)	C12—C11—H11	119.2
C3—C4—H4	120.5	C13—C12—C11	117.8 (4)
C5—C4—H4	120.5	C13—C12—H12	121.1
C4—C5—C6	120.6 (3)	C11—C12—H12	121.1
C4—C5—H5	119.7	C14—C13—C12	122.1 (4)
C6—C5—H5	119.7	C14—C13—Cl2	119.5 (3)
C1—C6—C5	119.5 (3)	C12—C13—Cl2	118.4 (3)
C1—C6—C7	123.0 (3)	C13—C14—C15	119.1 (3)
C5—C6—C7	117.3 (3)	C13—C14—H14	120.5
O1—C7—C8	120.8 (3)	C15—C14—H14	120.5
O1—C7—C6	121.0 (3)	C14—C15—C10	120.7 (4)
C8—C7—C6	118.2 (3)	C14—C15—H15	119.7
C9—C8—C7	122.4 (3)	C10—C15—H15	119.7
C9—C8—Br1	124.4 (3)		
			
C6—C1—C2—C3	−0.6 (5)	O1—C7—C8—Br1	31.2 (5)
C1—C2—C3—C4	1.4 (5)	C6—C7—C8—Br1	−151.1 (3)
C1—C2—C3—Cl1	−176.1 (3)	C7—C8—C9—C10	174.4 (4)
C2—C3—C4—C5	−1.3 (5)	Br1—C8—C9—C10	−1.5 (6)
Cl1—C3—C4—C5	176.2 (3)	C8—C9—C10—C11	165.5 (4)
C3—C4—C5—C6	0.2 (5)	C8—C9—C10—C15	−15.5 (7)
C2—C1—C6—C5	−0.4 (5)	C15—C10—C11—C12	−0.4 (6)
C2—C1—C6—C7	174.1 (3)	C9—C10—C11—C12	178.7 (3)
C4—C5—C6—C1	0.6 (5)	C10—C11—C12—C13	−2.0 (6)
C4—C5—C6—C7	−174.3 (3)	C11—C12—C13—C14	2.5 (5)
C1—C6—C7—O1	−149.5 (4)	C11—C12—C13—Cl2	−178.3 (3)
C5—C6—C7—O1	25.2 (5)	C12—C13—C14—C15	−0.5 (5)
C1—C6—C7—C8	32.8 (5)	Cl2—C13—C14—C15	−179.6 (3)
C5—C6—C7—C8	−152.5 (3)	C13—C14—C15—C10	−2.1 (5)
O1—C7—C8—C9	−145.1 (4)	C11—C10—C15—C14	2.5 (5)
C6—C7—C8—C9	32.6 (5)	C9—C10—C15—C14	−176.5 (4)

### Hydrogen-bond geometry (Å, °)

**Table:**

*D*—H···*A*	*D*—H	H···*A*	*D*···*A*	*D*—H···*A*
C1—H1···O1^i^	0.95	2.47	3.411 (5)	171
C11—H11···Cl1^ii^	0.95	2.81	3.619 (4)	143
C15—H15···Br1	0.95	2.69	3.377 (4)	129

Symmetry codes: (i) −*x*+1, *y*−1/2, −*z*+1; (ii) −*x*+1, *y*−1/2, −*z*+2.
